# A lightweight classification of adaptor proteins using transformer networks

**DOI:** 10.1186/s12859-022-05000-6

**Published:** 2022-11-04

**Authors:** Sylwan Rahardja, Mou Wang, Binh P. Nguyen, Pasi Fränti, Susanto Rahardja

**Affiliations:** 1 School of Computing, University of Eastern Finland, Joensuu, Finland; 2 School of Marine Science and Technology, Northwestern Polytechnical University and Singapore Institute of Technology, 710072 Xi’an, China; 3 Singapore Institute of Technology, Singapore, 138683 Singapore; 4 School of Mathematics and Statistics, Victoria University of Wellington, Wellington, New Zealand

**Keywords:** Adaptor protein, Protein classification, Deep learning, Transformer

## Abstract

**Background:**

Adaptor proteins play a key role in intercellular signal transduction, and dysfunctional adaptor proteins result in diseases. Understanding its structure is the first step to tackling the associated conditions, spurring ongoing interest in research into adaptor proteins with bioinformatics and computational biology. Our study aims to introduce a small, new, and superior model for protein classification, pushing the boundaries with new machine learning algorithms.

**Results:**

We propose a novel transformer based model which includes convolutional block and fully connected layer. We input protein sequences from a database, extract PSSM features, then process it via our deep learning model. The proposed model is efficient and highly compact, achieving state-of-the-art performance in terms of area under the receiver operating characteristic curve, Matthew’s Correlation Coefficient and Receiver Operating Characteristics curve. Despite merely 20 hidden nodes translating to approximately 1% of the complexity of previous best known methods, the proposed model is still superior in results and computational efficiency.

**Conclusions:**

The proposed model is the first transformer model used for recognizing adaptor protein, and outperforms all existing methods, having PSSM profiles as inputs that comprises convolutional blocks, transformer and fully connected layers for the use of classifying adaptor proteins.

## Background

Proteins make up a significant portion of the human body. This includes macroscopic structures like the musculoskeletal system, and microscopic processes such as cell to cell signaling. Due to its extensive role in human anatomy and physiology, it is no surprise that proteins contribute to a variety of pathologic conditions. For example, abnormalities of protein physiology result in multiorgan-involving diseases such as alpha-1 antitrypsin deficiency, cystic fibrosis and hereditary hemochromatosis. Even common conditions such as diabetes mellitus, with its established disorder in insulin, revolves around proteins. Hence, it is no surprise that elucidating protein structure and function is a key interest of the biomedical industry.

While communication is key in the first world setting, cells communicate at a microscopic level to maintain homeostasis by signal transduction. For accurate transmission of information, the signal must be conveyed reliably into the individual cells. Proteins play a key role in this process. Adaptor proteins are proteins with specific three-dimensional (3D) structural conformity that serve this purpose. Examples include MYD88 and SHC1. These adaptor proteins contain protein-binding molecules linking protein-binding partners together, facilitating the signal transduction cascade.

Due to its microscopic complexity, the study of protein structure has been limited until recent time. Proteins are synthesized via trinucleotide ribonucleic acid (RNA) codons, namely Adenosine, Uracil, Guanine and Cytosine. The triplet RNA codons, each of which could either be Adenosine, Uracil, Guanine or Cytosine, give rise to 64 distinct triple codons. The triplet codons each code for an amino acid. The RNAs are then transcribed into amino acids, the building blocks of proteins. Each amino acid is structurally different, and thus each protein will have a specific 3D structure to serve its unique function. Specifically in this context, adaptor proteins have structures to facilitate signal transduction. Due to coding overlaps, for example both CUU and CUC coding for amino acid leucine, the 64 permutations only code for 20 different amino acids.

Protein function prediction is an emerging field in bioinformatics [1], due to the availability of aforementioned databases and recent development in machine learning. Extensive research into protein structure and function resulted in the advent of databases such as UniProt [2] and Gene Ontology [3], kickstarting the drive into further protein structure research. Establishing the correct sequence for protein is vital in ensuring its 3D structure is intact. This explains the drive for protein function prediction research and the importance of minimizing losses or errors of amino acid sequences.

Since a minor discrepancy in amino acids could result in a distinct pathology, the accuracy of predictive methods is key. Satisfactory results have been achieved by prior studies such as position specific scoring matrix (PSSM) [4], biochemical properties (AAindex) [5], Pseudo Amino Acid Composition [6], and innovative methods using RNN and PSSM [7]. However, existing work still left much to be desired.

In the field of bioinformatics, application of deep learning algorithms such as CNN and RNN had been explored. In [7], RNN was used to model the sequence of PSSM. However, existing research had its limitations. The RNN has a set number of hidden state but the PSSM has a widely variable length. In contrast, transformer is a novel deep learning model that adopts the mechanism of attention [8]. It outperforms CNN and RNN in most cases, and can been used in genomics [9].

This paper aims to provide a new standard for distinguishing adaptor proteins. We hereby propose an ultra lightweight deep learning framework based on transformer and PSSM profiles to identify adaptor proteins. Transformer is a novel deep learning model for sequence analysis of adaptor proteins and the proposed model size in its optimum is only 1.6% of the state-of-the-art methods while in the sub-optimum the model size is less than 1% of the state-of-the-art, wherein both optimum and sub-optimum have better performance than previous best. It takes PSSM profile from the database as the input of the model, uses CNN and transformer for dimensionality reduction and sequence modeling, and outputs the probability of whether the protein under evaluation is an adaptor protein. We then considered usage of layer normalization and Gradient accumulation algorithm to solve the problem of single sample training caused by the variable length of proteins sequence. The experiment results on the independent dataset proved that our proposed model can effectively distinguish adaptor proteins from general proteins and exhibit superior performance compared to state-of-the-art algorithm.

## Results and discussion

### Dataset

We conducted our experiments on the dataset created in [7]. The dataset includes 1224 adaptor proteins and 11,078 non-adaptor proteins. We used all the protein sequences imported from two well-known databases, namely UniProt and Gene Ontology. Only protein sequences which have been published in papers (termed reviewed sequences) were selected. To prevent over-fitting, redundant sequences with sequence identity level of more than 30% were removed with the Basic Local Alignment Search Tool (BLAST) method [10].

We used one-fifths of both the adaptor proteins and the non-adaptor proteins as the test set to evaluate model performance. The rest of the valid sequences were used as a training dataset for model training. The detailed numbers of proteins are shown in Table 1.

**Table 1:** Dataset

	Train	Test	Total
Adaptor	1069	155	1224
Non-adaptor	9695	1383	11,078

### Settings

The Proposed model was implemented on NVIDIA GeForce 3090 GPU with PyTorch-lightning library. For all experiments, we trained the models for 50 epoches with Adam optimizer. The learning rate was initialized to $5\times 10^{-4}$, and halved if the Area Under the Curve (AUC) of validation set was not improved after 6 consecutive epochs. Early stopping was applied if the AUC was not improved after 20 consecutive epochs.

The batch size had to be set to 1 because of the problem of sequence length. Due to the batch size being set to 1, the gradient of model optimization would have been too random, making the model training unstable and difficult. Hence, to mitigate this issue, we used gradient accumulation. With gradient accumulation, the model variables would not be updated in every step until the gradients of a set number of batches were accumulated. In this article, the size of accumulate gradient batch is set to 24.

To evaluate the performance, we utilized fivefold cross-validation technique on the training dataset. We selected the model with the best performance on the validation set for each fold. Finally, the independent dataset was used to evaluate the model.

### Evaluation metrics

For simplicity of calculation and presentation, protein and non-adaptor protein are defined as positive data and negative data respectively. Common but effective evaluation metrics that were used to measure the classification performance of the proposed model, include accuracy, specificity, sensitivity and MCC (Matthew’s correlation coefficient), which can all be derived from the confusion matrix. In the confusion matrix, there are four categories, namely true positives, false positives, true negatives, false negatives, denoted as TP, FP, TN, FN respectively. Then the evaluation metrics are defined as follows:

1 $$\begin{aligned} {\textit{Accuracy}}\,=\, & {} \frac{{\textit{TP}}+{\textit{TN}}}{{\textit{TP}}+{\textit{TN}}+{\textit{FP}}+{\textit{FN}}} \end{aligned}$$

2 $$\begin{aligned} {\textit{Specificity}}\,=\, & {} \frac{{\textit{TN}}}{{\textit{TP}}+{\textit{FP}}} \end{aligned}$$

3 $$\begin{aligned} {\textit{Sensitivity}}\,=\, & {} \frac{{\textit{TP}}}{{\textit{TP}}+{\textit{FN}}} \end{aligned}$$

4 $$\begin{aligned} {\textit{MCC}}\,=\, & {} \frac{{\textit{TP}}\times {\textit{TN}}-{\textit{FP}}\times {\textit{FN}}}{\sqrt{({\textit{TP}}+{\textit{FP}})({\textit{TP}}+{\textit{FN}})({\textit{TN}}+{\textit{FP}})({\textit{TN}}+{\textit{FN}})}} \end{aligned}$$

In a binary classification, accuracy, specificity and sensitivity cannot reflect the real performance of the method, especially when the data is imbalanced. However, MCC is essentially a correlation coefficient between the observed and predicted binary classifications. Hence it is more used as a means to provide correlation information rather than accuracy of the classification, because it takes into account the balance ratios of the four confusion matrix categories.

Receiver Operating Characteristic (ROC) curve is also a common and reliable performance measurement for a classification problem at various thresholds settings. The AUC measures the entire two-dimensional (2D) area under the ROC curve. This score can reflect the performance of the classifier. The AUC value falls within a range from 0 to 1, where a higher value indicates a superior model. Besides, area under the precision-recall curve (AUPRC) is a useful performance metric for imbalanced data as well. In this paper, we focus on AUC and MCC.

### Comparison methods

Earlier, there were articles that utilize summation of amino acids to form 400-dimensional vector for the input of the neural networks [5, 11]. In addition, k-NN, Random Forest, Support Vector Machine (SVM), 2D Convolutional Neural Network and Recurrent Neural Networks (RNN) were also used to distinguish adaptor proteins [7]. RNN was being considered as state-of-the-art since it has the best performance as reported in [7] and achieved cross validation accuracy and MCC of 80.4% and 44.5% respectively. Specifically, the RNN model utilized PSSM profiles as inputs and obtained their features by two one-dimensional (1D) convolutional layers and 1D average pooling layers. In the model, the kernel size of convolution and pooling was 3, and the channel number of each distinct convolutional layer was 256. The features were then fed forward to Gated Recurrent Units (GRU) with 256 hidden cells. Lastly, the model processed the GRU output through a fully connected layer with 512 nodes, and then passed through a sigmoid layer to produce a prediction probability value.

Beside the RNN model, SVM and CNN [11] were also used to classify the adaptor proteins in [7]. CNN and RNN currently represent the state-of-the-art for protein classification problem. In the SVM, *g* was set to 0.5 and margin parameter *c* was set to 8. In CNN method, the filter number of convolution was 128 with kernel of $3\times 3$. In this article, we designed a transformer based system and compared against the CNN and RNN that currently represent the state-of-the-art for protein classification problem.

### Results

The proposed model utilized the newly introduced transformer blocks in combination with convolutional blocks and fully connected layers. In the simulation, the proposed model was compared with SVM method, CNN [11] and RNN [7], and the results in terms of sensitivity, specificity, accuracy, AUC and MCC were tabulated in Table 2 for both cross validation and independent tests. In addition, the model size is also shown as another metric of comparison. We observe that the proposed model achieved a higher AUC and MCC than all other existing methods. This proves that the sequential information of PSSM has more potential in classification of adaptor proteins, and the transformer based model can effectively extract and utilize it.

**Table 2:** Performance of adaptor proteins classification with different methods

Methods	Cross validation					Independent test					
	Sensitivity	Specificity	Accuracy	AUC	MCC	Sensitivity	Specificity	Accuracy	AUC	MCC	AUPRC
SVM	0.397	**0**.**934**	**0**.**881**	0.818	0.332	0.426	**0**.**932**	**0**.**881**	0.806	0.353	0.342
CNN [11]	0.801	0.738	0.750	0.834	0.368	0.851	0.780	0.787	0.874	0.423	0.437
RNN [7]	**0**.**812**	0.751	0.757	0.853	0.373	0.856	0.798	0.804	0.893	0.446	0.462
Proposed	0.786	0.803	0.801	**0**.**868**	**0**.**404**	**0**.**865**	0.827	0.831	**0**.**903**	**0**.**487**	**0**.**509**

Bold indicates the best value per metric

The sensitivity of the model reflects the effectiveness of a classifier in identifying true positives. The higher the sensitivity is, the more adapter proteins can be discovered from a sample of all proteins. From the result, we concluded that the sensitivity of the proposed method was significantly higher than SVM and CNN. In contrast, the model was only slightly better than RNN, because both the proposed method and RNN have the ability of sequence modeling. This shows that the sequence information plays an important role in identification of adaptor proteins.

Comparing RNN and our model, it was clear that our model is superior. As shown in Table 3, the model size of the proposed model is about 12.1k, which is only 1.6% of that of RNN. The FLOPs of the proposed method is about 216k, which is 2.5% of that of RNN. Despite being ultra lightweight, the model still achieved superior sensitivity, demonstrating that transformer is significantly more effective and efficient than CNN and RNN, as it allowed discovery of adaptor proteins more rapidly. In addition, the significantly reduced model size opens possibilities as it naturally makes embedding it into other platforms easier.

**Table 3:** Comparison of model size and complexity

Methods	Model size	FLOPs
CNN	160k	1629k
RNN [7]	729k	8549k
Proposed	**12**.**1k**	**216k**

Bold indicates the best value per metric

**Fig. 1:**
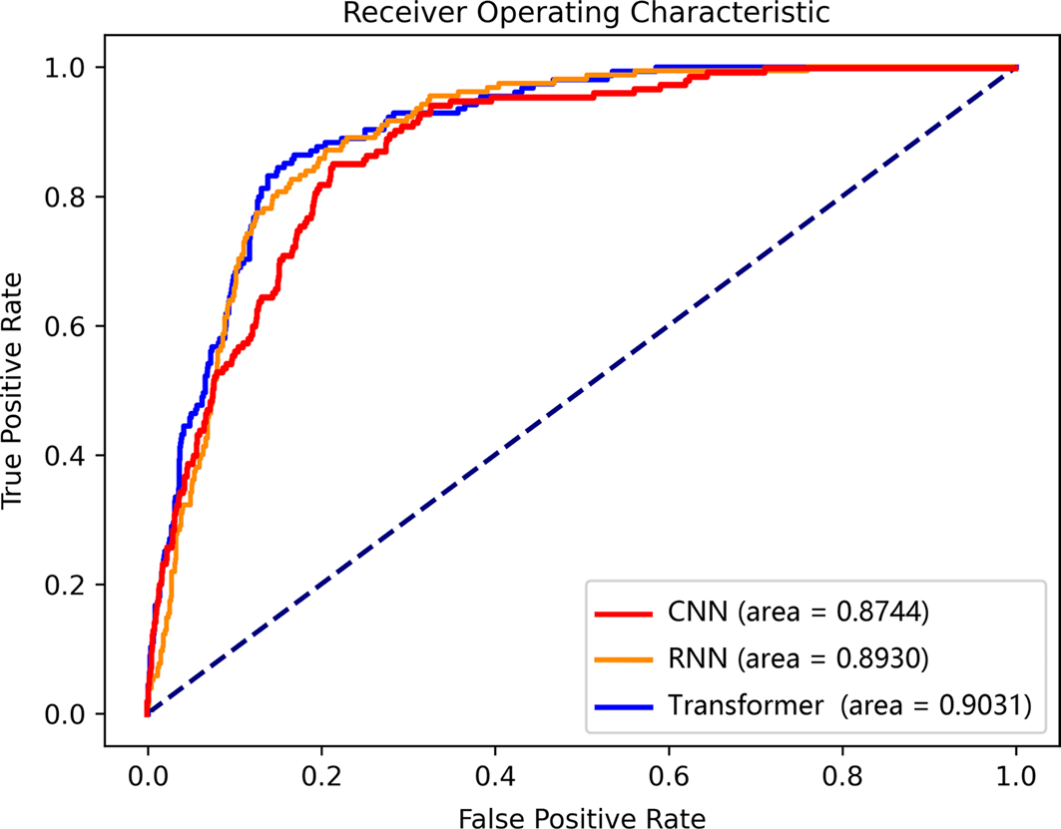
The receiver operating characteristic curve

Next, we consider the ROC curve as a comparison of efficacy. The ROC curve reflects the performance at all different decision threshold levels. The ROC curves of RNN and the tranformer based model are shown in Fig. 1. Evidently, the transformer model being tested outperforms RNN at most decision threshold levels. Moreover, the proposed method attains an AUC of 0.903 which shows that the model can still perform well despite the complication provided by varying sequence length in the dataset. Thus, this model is well suited to be used as adaptor proteins predictor.

To verify the effectiveness of transformer in the proposed method, we conducted ablation experiment by disabling the Transformer Encoder block. The result is shown in Table 4. From the Table, we can find that transformer can significantly improve the performance on all the metrics. Because transformer is utilized to explore sequence information, this ablation experiments demonstrate that the sequence information plays a significant role in identification of adaptor proteins again.

**Table 4:** Ablation studies on proposed method

Methods	AUC	MCC	AUPRC
Proposed (default)	0.903	0.487	0.509
w/o Transformer	0.889	0.441	0.459

Besides the self-attention, the Feed Forward Network (FFN) is also an important component in transformer as it can increase the complexity and improve performance. For comparison, we also performed experiments using different numbers of hidden nodes in transformer. The results are shown in Table 5. We observe that the system had the best performance when the FNN had 128 hidden nodes. As shown in Table 5, the lowest complexity with just 20 hidden nodes corresponds to a model size of 7.7k. This translates to less than 1% compared to the size of RNN based method, yet retaining its performance ability in terms of AUC and MCC.

**Table 5:** Performance of adaptor protein prediction on independent testing set using different numbers of hidden nodes in transformer. The model has three convolutional blocks with 20 convolution kernel

Hidden nodes	AUC	MCC	Model size
20	0.8941	0.4668	7.7k
32	0.9042	0.4696	8.2k
64	0.8978	0.456	9.5k
80	0.8998	0.4646	10.1k
128	**0**.**9031**	**0**.**4872**	12.1k
200	0.8999	0.4639	15.0k
256	0.8978	0.4633	17.3k

Bold indicates the best value per metric

## Conclusions

A new model to classify adaptor proteins is proposed in this article. The new model considers sequence information using transformer and PSSM profile. With this model, the PSSM feature was first obtained with Position-Specific Iterated BLAST (PSI-BLAST) method, then fed into a transformer based model for classification. It is the first time that transformer is utilized in the field of adaptor protein recognition, with clearly unparalleled results. The experimental results proved that the proposed method can achieve AUC of 0.903 and MCC of 0.487 on independent testing dataset, which triumphs the state-of-the-art methods. Despite a remarkably small size with just 1.6% of the previous leading model, this model demonstrated that transformers can model the sequence information of protein more effectively and efficiently than RNN based model.

With its multitude of functions, we hope our work in adaptor protein brings significant contribution to the field. This article shows that transformer based model can effectively model the sequence information, and we believe it can be further applied for detection, classification and analytics of other proteins functions, or even other challenges in bioinformatics and computational biology.

## Methods

### Proposed methods

**Fig. 2:**
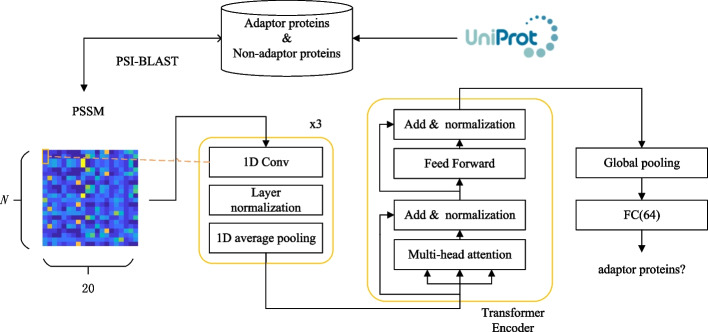
The flowchart of the proposed method

The objective of this study is to accurately identify adaptor proteins from an unclassified and unknown sequence. The flowchart is shown in Fig. 2. We first obtain the adaptor proteins and non-adaptor proteins from the database. Then, the processing contains two parts: the PSSM features were first extracted from proteins squence, then fed into a deep learning model to output a prediction. In the following, we introduce each step in detail.

### Feature extraction

As PSSM had shown promising results in bioinformatics research [4] in the past, it has since been a common and effective feature to describe protein secondary structure. A PSSM profile is a matrix which can be used to assimilate amino acid peptide sequences. The matrix is created by generating two sequences with different peptide sequences, but comparable with 3 dimensional conformation. Given that there are 20 distinct amino acids, we simply use a $N\times 20$ matrix, where *N* denotes the sequence of interest. The individual components of the PSSM profile can be denoted as $P_{ij}$, where *i* represents the amino acid in the *j*-th position of the sequence. A high output value is optimal, as it means the peptide sequence is conserved, while a negative value is suboptimal and represents a compromised value. In this study, we utilized protein sequences for dataset in FASTA format. Then, PSI-BLAST was used to change FASTA sequences into PSSM profiles.

A significant challenge posed by data of proteins sequence is a wide range of sequence length. For example, in this work, the shortest sequence of PSSM profile has only 18 points, but the longest sequence of PSSM has more than 20,000 points. The variation in length brought about challenges in establishing a reliable model, as most models require input sequences of similar length. Although some deep learning models can process vectors with variable length, the input sequences should ideally have equal length during the training stage to build a reliable model. To tackle this issue, some authors consider the following solution [12]: We could sum all the amino acids in PSSM profile, and PSSM profiles with $N\times 20$ is converted into a $20\times 20$. While the input length was a constant, this came at a cost of loss of sequence information as the ordering of the PSSM profile is compromised.

Protein sequences are distinct from other sequence analysis problems. By nature, protein sequences are distinct from other topics such as speech and text. In most applications such as audio processing, there were common methods proposed to solve this problem such as padding, sliding windows and etc. The methods which are effective for speech and text are unable to achieve similar results for protein sequences. Padding makes short sequences meaningless when standard deviation of sequence length is large, and sliding window will break the protein sequences leading to artificial and meaningless sequences. Similar to [7], we had to treat each entire protein sequence as a whole and input the sequence into the model. This led to the batch size preset of the input model to 1.

### Models

As shown in Fig. 2, the model consists of three modules: three convolutional block, transformer and fully connected layers.

The model took PSSM profiles as inputs and extracted their features by three convolutional blocks, namely 1D convolutional layers and 1D average pooling layer, where the 1D convolution operates on the sequence dimension. Then, the extracted features were fed into the transformer, where the spatial context within the entire PSSM profile was explored and modeled. Subsequently, global pooling was used to summarize the sequence and achieve a 1D vector. The advantage of global pooling was the ability to map the sequence with different length into a vector with the same length. Hence, we used global average pooling.

In the final stage, two fully-connected (FC) layer and a sigmoid function were used to classify the vector. The RNN model output is a scalar having [0, 1] which indicates the probability of the testing PSSM profile belonging to the adaptor or non-adaptor protein categories. Finally, to avoid overfitting, dropout of 0.5 was applied after the first FC layer.

### Convolutional block

CNN is a powerful and effective method for feature transformation. Comparing to traditional and manually designed features, the learnable feature extracted by CNN is more compact and effective. Therefore, we used CNN to further extract more effective features from PSSM before sequence modeling.

In CNN, the features are converted into a higher dimension feature map with a set of convolution kernels. To obtain good feature representation, some incorporate more convolution kernals as high as 256 [7]. Because transformer has a strong ability of sequence modeling, the requirement of convolution kernels for good feature representation can be reduced.

In addition, a large feature map will increase memory consumption of transformers. Therefore, we propose the usage of three convolution layers with only 20 convolution kernels of $3\times 3$, followed by a normalization layer. Then, the 1D average pooling layer with kernel of 2 was applied which was essentially aimed to lessen the feature maps dimension and at the same time enlarge the receptive field of the CNN network.

Batch normalization is a common method in CNN. It applies scalar scale and bias for all batches. It can make the convergence of CNN model more stable and rapid during the training, and reduce the undesired effect of model over-fitting. However, batch normalization was not applicable in this work because the batch size had to be 1. To address this issue, layer normalization was used. Unlike batch normalization, layer normalization applies per-element scale and bias along the channel dimension [13]. Given the feature map *x*, the layer normalization can be expressed as

5 $$\begin{aligned} y = \frac{x-E[x]}{\sqrt{{\textit{Var}}[x]}}*\gamma +\beta \end{aligned}$$

where *E* and ${\textit{Var}}$ denote expectation and variance respectively, and $\gamma$ and $\beta$ are learnable affine transform parameters.

### Transformer

Transformer is a novel neural network for natural language processing, first proposed by Google [8]. Transformer has advantages of both sequence modeling like RNN and parallel processing like CNN. With its self-attention mechanism, transformer can explore longer contextual information than RNN. Therefore, it has been rapidly applied in various fields such as machine translation [14], speech [15], image [16] and genome [17].

The transformer architecture is essentially an encoder-decoder model [18]. While the encoder has encoding layers that process input systemically, the decoder has decoding layers with similar function based on the encoding layer output. Both share structurally similar model. However, the decoder is dependent on encoding output. Specifically, we focused on the transformer encoders in this article. It consists of three core modules: Scale dot-product attention, multi-head attention and position-wise FFN.

**Fig. 3:**
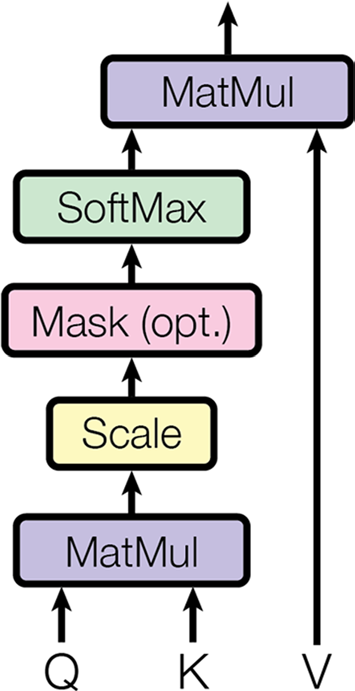
Scaled dot-product attention. This figure is copied from [8]

**Fig. 4:**
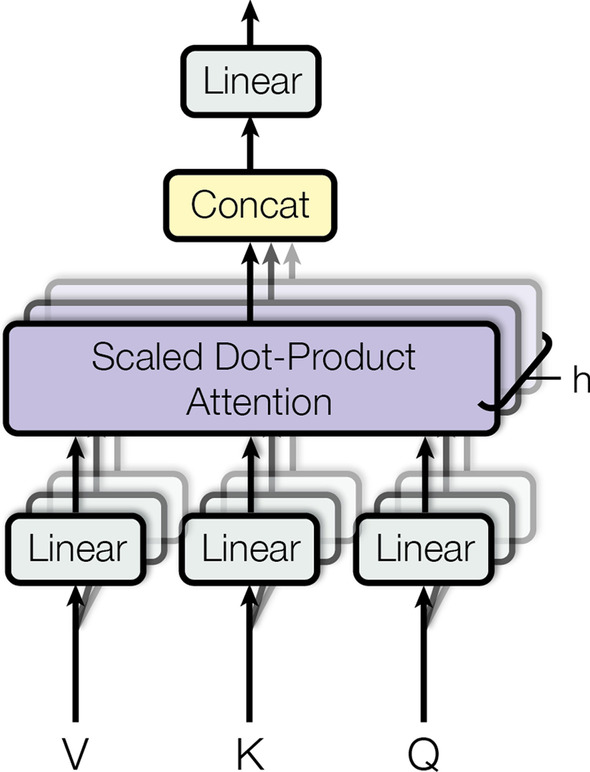
Multi-head attention. This figure is copied from [8]

The most basic element in a transformer is the scaled dot-product attention, which is essentially a self-attention mechanism that aims to efficiently combine different positions of input sequences so as to generate inputs representations, as shown in Fig. 3. Each individual output has a significance value which is attained from the attention function derived from query of the respective keys and adding the weighted sum to these outputs would produce the outcome of the transformer module. As shown in Fig. 4, multi-head attention comprises multiple scaled dot-product attention modules. In the first stage, the module linearly calculated the inputs h times with varying and learnable linear projections to acquire parallel queries, keys and values respectively. In the subsequent stage, dot-product attention was then applied to these queries, keys and values together.

Position-wise FFN is a completely integrated feed-forward networking. It consists of two linear transformations with a ReLU activation in the middle. Besides these three core modules, transformers incorporate multiple residual and normalization layers, with layer normalization employed [19]. The overall architecture of the transformer can be mapped as:

6 $$\begin{aligned} Q_i\,=\,ZW_i^Q,K_i\,=\,ZW_i^K,V_i\,=\,ZW_i^V,i\in [1,h] \end{aligned}$$

7 $$\begin{aligned} {\textit{head}}_i\,=\, {\textit{Attention}}(Q_i,K_i,V_i) \end{aligned}$$

8 $$\begin{aligned}\,=\, {\textit{softmax}}(\frac{Q_iK_i^T}{\sqrt{d}})V_i \end{aligned}$$

9 $$\begin{aligned} {\textit{MultiHead}}\,=\, {\textit{Concat}}({\textit{head}}_1,\ldots ,{\textit{head}}_h)W^O \end{aligned}$$

10 $$\begin{aligned} {\textit{Mid}}\,=\, {\textit{LayerNorm}}(Z+{\textit{MultiHead}}) \end{aligned}$$

11 $$\begin{aligned} {\textit{FFN}}\,=\, {\textit{ReLU}}({\textit{MidW}}_1+b_1)W_2+b_2 \end{aligned}$$

12 $$\begin{aligned} {\textit{Output}}\,=\, {\textit{LayerNorm}}({\textit{Mid}}+{\textit{FFN}}) \end{aligned}$$

Here, $Z\in R^{l\times d}$ is the input with length *l* and dimension *d*, and $Q_i,K_i,V_i\in R^{l\times d/h}$ are the mapped queries, keys and values respectively. $W_i^Q, W_i^K, W_i^V\in R^{d\times d/h}$ and $W^O\in R^{d\times d}$ are parameter matrices. FFN denotes the output of the position-wise FFN, in which $W_1\in R^{d\times d_{ff}}, W_2\in R^{d_{ff}\times d},b_1\in R^{d_{ff}},b_2\in R^d$. In this work, *d* was set to 20, *h* was set to 5, and $d_{ff}$ was set to 128.

### Loss

Based on the provided dataset, there were obvious discrepancies in the available adaptor proteins versus non adaptor protein, where the latter significantly outnumbered the former. Hence, we utilized weighted binary cross-entropy loss in the training. Let *x* denote the input sequence, *y* denote label , *w* denote the weight, *L* denote the loss, we have the following equation

13 $$\begin{aligned} L \,=\, w*y*\log x + (1-y)*\log (1-x), \end{aligned}$$

where weight *w* is set to the inverse class frequency. In this work, it is set to [10.07, 1.11].

## Data Availability

The datasets analysed during the current study are available at https://github.com/ngphubinh/adaptors. Our source code are available at https://github.com/wangmou21/adaptor. If someone wants to request the raw data or source code, please feel free to contact Mou Wang or Susanto Rahardja.

## Abbreviations

AUC: Area under the ROC curve
CNN: Convolutional neural network
FFN: Feed forward network
MCC: Matthew’s correlation coefficient
PSSM: Position specific scoring matrix
ReLU: Rectified linear unit
RNN: Recurrent neural network
ROC: Receiver operating characteristic
SVM: Support vector machine
