# Correction: A lightweight classification of adaptor proteins using transformer networks

**DOI:** 10.1186/s12859-022-05131-w

**Published:** 2023-01-11

**Authors:** Sylwan Rahardja, Mou Wang, Binh P. Nguyen, Pasi Fränti, Susanto Rahardja

**Affiliations:** 1 School of Computing, University of Eastern Finland, Joensuu, Finland; 2 School of Marine Science and Technology, Northwestern Polytechnical University, Xi’an, 710072 China; 3 Singapore Institute of Technology, Singapore, 138683 Singapore; 4 School of Mathematics and Statistics, Victoria University of Wellington, Wellington, New Zealand

**Correction to: BMC Bioinformatics (2022) 23:461** 10.1186/s12859-022-05000-6

Following the publication of the original article [1], the authors identified an error in affiliation 2.

The incorrect affiliation is: 2 School of Marine Science and Technology, Northwestern Polytechnical University and Singapore Institute of Technology, 710072 Xi’an, China.

The correct affiliation is: 2 School of Marine Science and Technology, Northwestern Polytechnical University, 710072 Xi’an, China.

The original article [1] has been corrected.

## References

[1] Rahardja S (2022). A lightweight classification of adaptor proteins using transformer networks. BMC Bioinform.

